# A Novel Audiovisual P300-Speller Paradigm Based on Cross-Modal Spatial and Semantic Congruence

**DOI:** 10.3389/fnins.2019.01040

**Published:** 2019-09-27

**Authors:** Zhaohua Lu, Qi Li, Ning Gao, Jingjing Yang, Ou Bai

**Affiliations:** ^1^School of Computer Science and Technology, Changchun University of Science and Technology, Changchun, China; ^2^Department of Electrical and Computer Engineering, Florida International University, Miami, FL, United States

**Keywords:** brain–computer interface, audiovisual, P300-speller, spatial congruence, semantic congruence

## Abstract

**Objective:**

Although many studies have attempted to improve the performance of the visual-based P300-speller system, its performance is still not satisfactory. The current system has limitations for patients with neurodegenerative diseases, in which muscular control of the eyes may be impaired or deteriorate over time. Some studies have shown that the audiovisual stimuli with spatial and semantic congruence elicited larger event-related potential (ERP) amplitudes than do unimodal visual stimuli. Therefore, this study proposed a novel multisensory P300-speller based on audiovisual spatial and semantic congruence.

**Methods:**

We designed a novel audiovisual P300-speller paradigm (AV spelling paradigm) in which the pronunciation and visual presentation of characters were matched in spatial position and semantics. We analyzed the ERP waveforms elicited in the AV spelling paradigm and visual-based spelling paradigm (V spelling paradigm) and compared the classification accuracies between these two paradigms.

**Results:**

ERP analysis revealed significant differences in ERP amplitudes between the two paradigms in the following areas (AV > V): the frontal area at 60–140 ms, frontal–central–parietal area at 360–460 ms, frontal area at 700–800 ms, right temporal area at 380–480 and 700–780 ms, and left temporal area at 500–780 ms. Offline classification results showed that the accuracies were significantly higher in the AV spelling paradigm than in the V spelling paradigm after superposing 1, 2, 5, 6, 9, and 10 times (*P* < 0.05), and there were trends toward improvement in the accuracies at superposing 3, 4, 7, and 8 times (*P* = 0.06). Similar results were found for information transfer rate between V and AV spelling paradigms at 1, 2, 5, 6, and 10 superposition times (*P* < 0.05).

**Significance:**

The proposed audiovisual P300-speller paradigm significantly improved the classification accuracies compared with the visual-based P300-speller paradigm. Our novel paradigm combines spatial and semantic features of two sensory modalities, and the present findings provide valuable insights into the development of multimodal ERP-based BCI paradigms.

## Introduction

Brain–computer interfaces (BCIs), which provide a direct method of communication between the brain and external devices (Kubler et al., 2001; Wolpaw et al., 2002), can help severely disabled people, especially patients with amyotrophic lateral sclerosis, to interact with the outside world (Kubler and Neumann, 2005; Nijboer et al., 2008). The P300-speller system is one of the most commonly used BCI applications. P300 refers to an event-related potential (ERP), which occurs around 300 ms after the presentation of a stimulus and is elicited by an oddball event. In the P300-speller system, the user focuses on the desired character (i.e., the target character) and counts the number of times of its intensification; the probability of the target character highlighted each time is small, and this oddball event would elicit a P300 potential. The P300-speller system outputs the target character by detecting the P300 potential; thus, it realizes communication with the outside world by the way of “mental typewriting” (Farwell and Donchin, 1988; Bernat et al., 2001). In the past few decades, studies have investigated many P300-speller systems, including the auditory-based P300-speller (Guo et al., 2010; Halder et al., 2010; Xu et al., 2013), tactile-based p300-speller (Brouwer and Van Erp, 2010; Van der Waal et al., 2012), and visual-based P300-speller (Kaufmann et al., 2011; Jin et al., 2014; Li et al., 2015; Xu et al., 2018). The visual-based P300-speller is the most common P300-speller because its performance is much better than those of the other two types (Belitski et al., 2011; An et al., 2014; Wang et al., 2015; Hammer et al., 2018). However, the visual P300-speller system is still in an exploratory stage because its accuracy and information transfer rates (ITRs) are not satisfactory for practical application. In addition, the visual P300-speller is limited for some patients with neurodegenerative diseases, in which muscular control of the eyes may be impaired or deteriorate with time (Szmidt-Salkowska and Rowinska-Marcinska, 2005; Suminski et al., 2009). Hence, it is necessary to design a multimodal P300-speller based on audiovisual stimuli that is superior to visual-based P300-spellers and can be used more universally.

Some recent studies have investigated the audiovisual P300 BCI systems. Sellers and Donchin (2006) evaluated the effectiveness of a P300-based BCI system involving bimodal audiovisual stimuli and found that its performance was not significantly better than that of the unimodal visual mode system. Belitski et al. (2011) proposed an audiovisual P300-speller that was implemented by combining the row/column number with a spoken number and demonstrated that the effectiveness of the modified audiovisual P300-speller slightly out-performed that of either the visual-based or the auditory-based P300-speller. These findings provide a basis for further exploration of the audiovisual bimodal P300-speller.

The spatial and semantic information of auditory and visual stimuli may affect the integration of the auditory and visual features of these stimuli (Stein and Meredith, 1993). For instance, semantically congruent audiovisual stimuli elicit larger amplitudes between 180 and 210 ms than do audiovisual stimuli without semantic information (Hu et al., 2012) and significantly enhance behavioral performances compared with unimodal visual and auditory stimuli (Laurienti et al., 2004). A functional magnetic resonance imaging (fMRI) study has demonstrated that neural responses are more pronounced for semantically matching audiovisual stimuli than for unimodal visual and auditory stimuli in the multisensory superior temporal sulcus areas (Hein et al., 2007). In addition, ERP amplitudes elicited by multisensory stimuli (i.e., audiovisual stimuli) are larger than the sum of the amplitudes elicited by visual and auditory stimuli at 200–220 ms (central–medial positivity) and 300–450 ms (centro–medial positivity) when the spatial orientation of the auditory and visual stimuli is congruent (Talsma and Woldorff, 2005). Audiovisual stimuli combining spatial and semantic information also influence audiovisual integration. When the auditory sound sources are spatially congruent with the semantically matching visual stimulus, the right middle and superior temporal gyrus areas are more activated, which indicates that spatial congruency appears to enhance the semantic integration of audiovisual stimuli (Plank et al., 2012).

In the present study, we designed a novel audiovisual P300-speller paradigm (AV spelling paradigm) based on spatial and semantic congruence to achieve spelling of multiple characters (i.e., multiple classifications). We compared the performance of the audiovisual P300-speller with that of a unimodal visual P300-speller (V spelling paradigm).

## Materials and Methods

### Subjects

Eighteen subjects (nine males) aged 19–29 years (mean age, 24.8 ± 2.27 years) were recruited in this study. One subject had previously participated in similar BCI studies. These subjects did not have any known neurological disorders and had normal or corrected-to-normal vision and normal hearing. They were undergraduates or master students and were familiar with the alphabet used in this study. They provided written informed consent in accordance with the Declaration of Helsinki, allowed use of the data, and acknowledged their personal rights. All subjects were compensated with 100 RMB after completion of the experiments. The study was approved by the ethics committee of the Changchun University of Science and Technology.

### Stimuli and Paradigms

**FIGURE 1:**
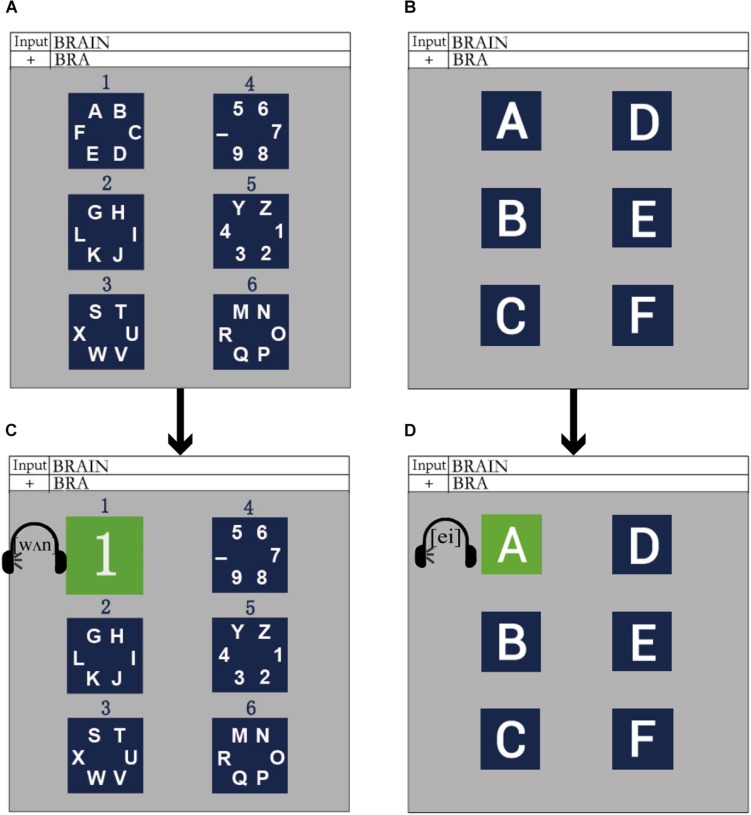
The stimuli and experimental paradigm in the audiovisual P300-speller. **(A)** The layout of level 1 in the AV spelling paradigm; **(B)** an example for the layout of level 2, which corresponded to group-area 1 in level 1 of the AV spelling paradigm; **(C)** an example for a group-area intensified in level 1 of the AV spelling paradigm; **(D)** an example for a sub-area highlighted in level 2 of the AV spelling paradigm.

The spatially and semantically congruent audiovisual P300-speller paradigm (AV spelling paradigm) was proposed on the basis of the traditional regional flashing spelling paradigm (Fazel-Rezai et al., 2011) and included two levels: level 1 consisted of several character group-areas and level 2 consisted of single characters from one of the group-areas in level 1, each of which was treated as a sub-area. The stimuli and experimental paradigm designs are shown in Figure 1. We divided 36 characters into six group-areas (i.e., level 1), and the six group-areas were arranged in the left and right columns (three in each column). The purpose of this arrangement was to match the pronunciation of the left and right characters with the left and right channels. In order to locate each group-area, we numbered the six group-areas from top to bottom and from left to right, i.e., 1–6 (Figure 1A). There were six sub-areas in level 2, which corresponded to six characters in a group-area. Similarly, the six sub-areas were arranged in the left and right columns (three in each column). Figure 1B shows the layout of level 2, which corresponds to group-area 1 in level 1. To induce obvious ERPs and make the subject feel comfortable, we chose blue as the background color for the group-area and sub-areas (Takano et al., 2009).

The design of the audiovisual P300 spelling paradigm with spatial and semantic congruence was as follows: when a group-area on the left was highlighted (e.g., number 1), it was covered by the corresponding number on a green background (Takano et al., 2009), during which the pronunciation of the corresponding group-area number was played simultaneously in the left earphone with a maximum sound-pressure level of approximately 65 dB (Senkowski et al., 2007; Figure 1C); when a group-area on the right was highlighted (e.g., number 4), it was covered with the corresponding number on a green background, and the pronunciation of the corresponding group-area number was played in the right earphone at the same time. Consequently, the spatial and semantic congruence of the group-area was ensured. After a group-area was selected, it transformed to level 2 (i.e., the sub-area), which was the spread of a selected group-area. When a sub-area on the left (or the right) was highlighted (e.g., character “A,” Figure 1D), the sub-area was covered with the corresponding character on a green background, and the pronunciation of the corresponding character was played in the left (or right) earphone at the same time. Thus, the spatial and semantic congruence of the sub-area was also ensured.

The control paradigm was a unimodal visual P300-speller (V spelling paradigm), in which the presentation of stimuli was the same as in the AV spelling paradigm, except that there was no sound.

### Experimental Procedure

**FIGURE 2:**
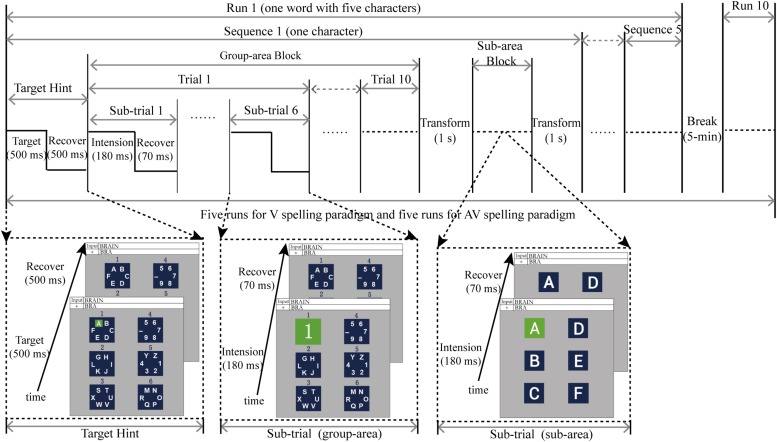
Time course of the experiment.

The experiment was conducted in a shielded room that was dark and soundproof. After completing the preparation for EEG recording, subjects sat comfortably in front of the monitor, and their eyes were about 70 cm from the computer monitor. Subjects were familiar with the experimental task prior to commencement, and they were asked to avoid blinking during the stimulus presentation. To specify the target character for a subject’s output, the target character with a green background was first presented for 500 ms by audiovisual synchronization (Figure 2), and the background was then presented for 500 ms (Figure 2). Subsequently, the six group-areas began to flash in a pseudo-random order. The stimulus onset asynchrony (SOA) of the flashing group-area was 250 ms, in which each group-area was highlighted for 180 ms before reverting to the background for 70 ms. The process was referred to as a sub-trial. In a trial, each group-area flashed once (a total of six sub-trials). The trial was repeated 10 times, making up a block (Figure 2). After completing a block for a group-area, it reverted to the background for 1 s. The display was then transformed to level 2, and the sub-area flashed in a manner similar to that of the group-area. After the sub-area flashed, it returned to the background for 1 s again before the next target character was presented; the process was then repeated. A sequence consisted of a group-area block and a sub-area block to output a target character (Figure 2). The output of a word with five characters was defined as a run. Between each run, subjects were permitted a 5-min break. Each subject took part in five runs (five words) for each spelling paradigm (AV and V spelling paradigms), and a total of 10 runs were presented in a pseudorandom order to avoid learning effects.

### Data Acquisition and Processing

#### Data Acquisition

**FIGURE 3:**
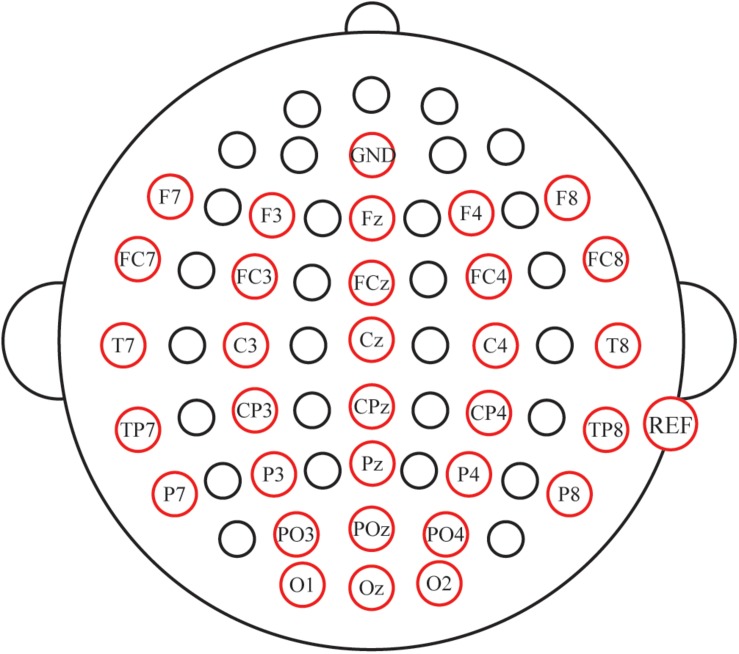
Electrode locations for the study.

Electroencephalogram (EEG) signals were recorded with a NeuroScan amplifier (SynAmps 2, NeuroScan Inc., Abbotsford, Australia) from 31 Ag/AgCl scalp electrodes (F7, F3, Fz, F4, F8, FC7, FC3, FCz, FC4, FC8, T7, C3, Cz, C4, T8, TP7, CP3, CPz, CP4, TP8, P7, P3, Pz, P4, P8, PO3, POz, PO4, O1, Oz, and O2; Figure 3). The AFz was used as a ground, and the reference electrode was placed on the mastoid of the right ear. Vertical and horizontal eye movements were measured using the VEO and HEO electrodes, respectively. The impedance was maintained below 5 KΩ. All signals were digitized at a rate of 250 Hz. EEG data were digitally filtered with a band-pass filter of 0.01–100 Hz. Presentation of the auditory and visual stimuli was controlled by the E-prime 2.0 software (PST Inc., Savannah, GA, United States).

#### Data Preprocessing

Original EEG data were first corrected for ocular artifacts using a regression analysis algorithm (Semlitsch et al., 1986) and were digitally filtered using a band-pass filter of 0.01–30 Hz. Data were then divided into epochs from 100 ms before the onset of each stimulus to 800 ms after the onset, and baseline corrections were made against −100–0 ms. Bad stimuli were removed by setting ±80 μV as the threshold for ocular artifacts. ERP data were averaged for each stimuli type (target, non-target stimulus) and used for the ERP waveform analysis. Grand-averaged ERP data were acquired from all subjects for each stimulus type in the two spelling paradigms (AV and V spelling paradigms). The pre-processed data, including segmentation, baseline correction, removal of bad stimulus, and filtering, were used for feature extraction and classification.

#### Feature Extraction and Classification Scheme

For the P300-speller, feature extraction for classification is based on temporal and spatial features of EEG data. For the temporal feature, we selected the time window in which there were obvious ERP amplitudes elicited by target stimuli and those with differences between target and non-target stimuli. Spatial features depended on the electrodes. The *r*^2^-values can provide the mathematic foundation for selecting channels (electrodes) and features of each channel. The *r*^2^ is calculated by formula (1)

(1) $$r^{2}={\left(\frac{\sqrt{N_{1}\,N_{2}}\,\left(\text{mean}\,\left(x_{1}\right)-\text{mean}\,\left(x_{2}\right)\right)}{\left(N_{1}+N_{2}\right)\,\text{std}\,\left(x_{1}\,⋃x_{2}\right)}\right)}^{2},$$

where *N*_1_ and *N*_2_ represent the sample sizes of the target and non-target, respectively; *x*_1_ and *x*_2_ are the feature vectors of target and non-target, respectively.

The EEG was then down-sampled from 250 to 50 Hz by selecting every five samples from the epoch. Thus, the size of the feature vector was *C_*N*_* × *P*_*N*_ (*C*_*N*_ represents the number of channels, and *P*_*N*_ represents the sample points).

Bayesian linear discriminant analysis (BLDA) was used to classify the EEG data. BLDA is an extension of Fisher’s linear discriminant analysis (FLDA) that helps avoid overfitting. The details of the algorithm can be found in a previous report (Hoffmann et al., 2008). We used fivefold cross-validation to calculate the individual accuracy in the offline experiment.

### Information Transfer Rate

Information transfer rate is generally used to evaluate the communication performance of a BCI system and is a standard measure that accounts for accuracy, the number of possible selections, and the time required to make each selection (Thompson et al., 2013). ITR (bits min^–1^) can be calculated as:

(2) $$\text{ITR}=\frac{60\,\left(P\,\log_{2}\left(P\right)+\left(1-P\right)\,\log_{2}\frac{1-P}{N-1}+\log_{2}N\right)}{T},$$

where *P* denotes the probability of recognizing a character, *T* is the time taken to recognize a character, and *N* is the number of classes (*N* = 36).

### Data Analysis

Differences in the waveforms between the V and AV spelling paradigms were analyzed using a one-way repeated measures ANOVA with two within-subject factors, i.e., spelling paradigm (V spelling paradigm and AV spelling paradigm) and electrode (the choice of the electrode was based on the difference of ERP waveforms elicited by target stimuli). The latencies of P3a at Fz and P3b at Pz were calculated in the V and AV spelling paradigms, and a pairwise *T*-test was conducted to analyze the latency difference between them. *T*-tests were also conducted to compare the accuracies and ITRs at each superposition time in the V and AV spelling paradigms (superposition time represents the time of repeated intensification of the six group-areas/sub-areas, and one superposition time is composed of one trial of group-area and one trial of sub-area). In addition, false discovery rate (FDR) correction was performed for multiple comparisons. Statistical analyses were conducted using SPSS version 19.0 (IBM Corp., Armonk, NY, United States).

## Results

### ERP Results

**FIGURE 4:**
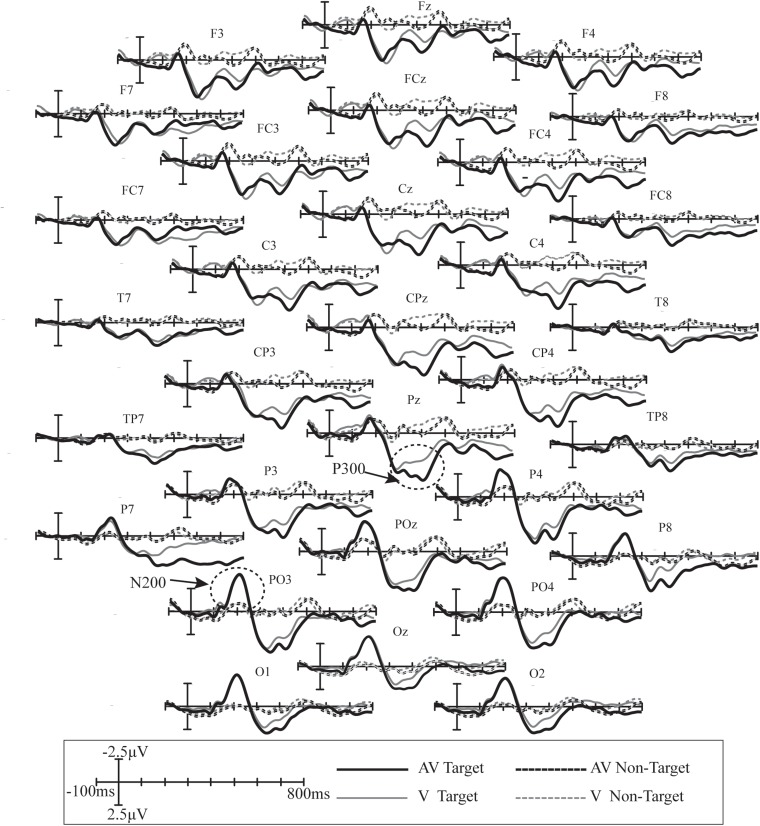
Superimposed grand-averaged ERPs elicited by target and non-target stimuli at 31 electrodes in the V and AV spelling paradigms. AV target, the ERP waveform elicited by the audiovisual target stimuli; AV non-target, the ERP waveform elicited by the audiovisual non-target stimuli; V target, the ERP waveform elicited by the visual target stimuli; V non-target, the ERP waveform elicited by the visual non-target stimuli.

Mean ERP waveforms were calculated across all subjects from 31 electrodes in the V and AV spelling paradigms (Figure 4). Clear positive deflections in the waveform along with two clear peaks were observed between 200 and 500 ms at F3, Fz, F4, FC3, FCz, FC4, C3, Cz, C4, CP3, CPz, CP4, P3, Pz, P4, PO3, POz, PO4, O1, Oz, and O2, which may be P300 potential. In addition, a clear negative waveform was observed at approximately 200 ms at P7, P3, Pz, P4, P8, PO3, POz, PO4, O1, Oz, and O2, which may be N200 potential.

**FIGURE 5:**
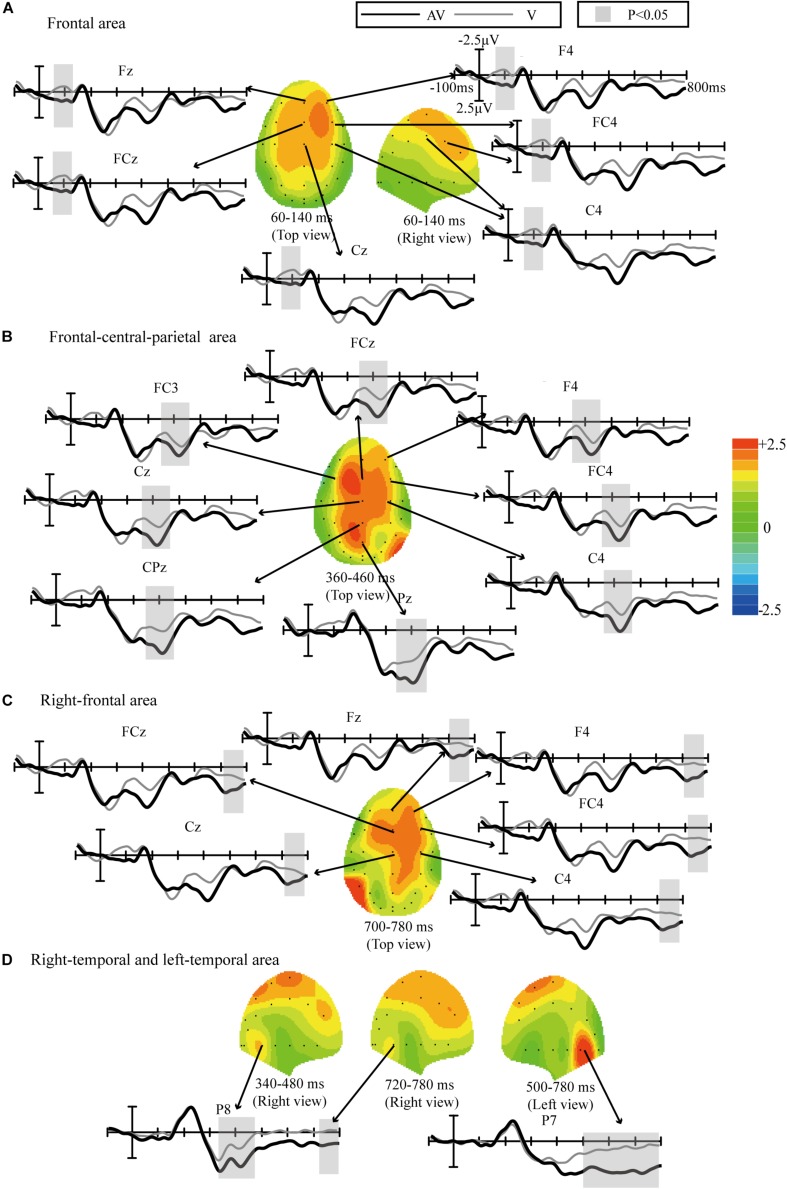
Comparison of waveforms elicited by the target stimuli in the V and AV spelling paradigms and scalp topographies from waveforms with difference formed by subtracting ERPs of the V spelling paradigm from those of the AV spelling paradigm: **(A)** the frontal area at 60–140 ms; **(B)** frontal–central–parietal at 360–460 ms; **(C)** right frontal at 700–780 ms; and **(D)** right temporal at 340–480 and 720–780 ms, and left temporal at 500–780 ms.

Scalp topographies were obtained by subtracting the ERP waveforms elicited by the target stimuli in the V spelling paradigm from those elicited in the AV spelling paradigm; time-domain features with statistically significant differences in the waveforms were then analyzed based on these scalp topographies, and the results were corrected by FDR (Figure 5). Statistically significant differences between the AV and V spelling paradigms were observed in the waveforms as follows: (1) 60–140 ms at frontal area [*F*(1,17) = 10.642, *P* < 0.005] (Figure 5A); (2) 360–460 ms at the frontal–central–parietal areas [*F*(1,17) = 11.921, *P* < 0.002] (Figure 5B); (3) 700–780 ms at the right frontal areas [*F*(1,17) = 6.031, *P* < 0.05] (Figure 5C); and (4) 340–480 [*F*(1,17) = 4.743, *P* < 0.05] and 720–780 ms [*F*(1,17) = 4.021, *P* < 0.05] at the right temporal areas and 500–780 ms at the left temporal areas [*F*(1,17) = 15.16, *P* < 0.001] (Figure 5D).

**FIGURE 6:**
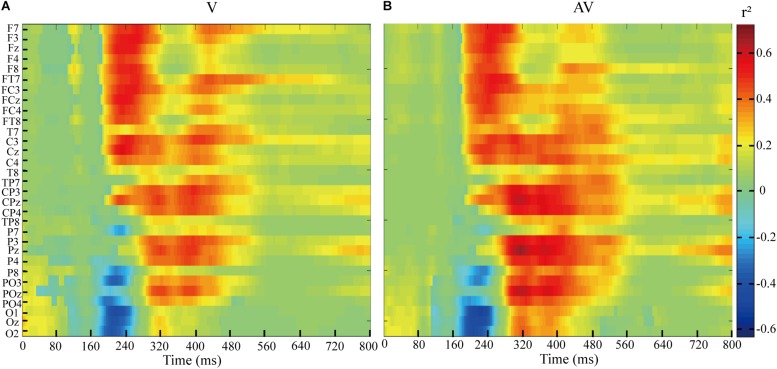
*r*^2^-values of ERPs amplitudes elicited by target and non-target stimuli at 0–800 ms based on EEG data of all subjects in the AV and V spelling paradigms. **(A)** *r*^2^-values of ERPs for the V spelling paradigm. **(B)** *r*^2^-values of ERPs for the AV spelling paradigm.

The feature differences between target and non-target stimuli in V and AV spelling paradigms were indicated by the *r*^2^-values (Figure 6). As shown in Figure 6, the feature differences of ERPs between target and no-target stimuli were mainly found between 200 and 320 ms at F7, F3, Fz, F4, F8, FT7, FC3, FCz, FC4, FT8, C3, Cz, and C4 electrodes and between 300 and 560 ms at CP3, CPz, CP4, P3, Pz, P4, PO3, POz, and PO4 electrodes in V and AV spelling paradigms. In addition, the feature differences of ERPs between target and no-target stimuli at 300-560 ms at CP3, CPz, CP4, P3, Pz, P4, PO3, POz, and PO4 electrodes were larger in the AV spelling paradigm than in the V spelling paradigm. In order to present positive and negative deflections of ERP amplitude and reflect richer information by the graph, we set the *r*^2^-value corresponding to the negative ERP amplitude value as a negative value.

### Latency

**FIGURE 7:**
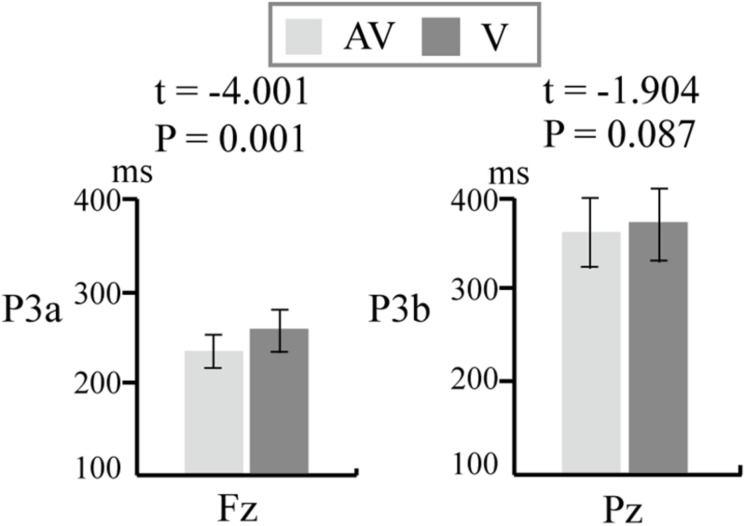
Comparison of latency (means ± SEM) of P3a at Fz and P3b at Pz between the V and AV spelling paradigms. V, visual spelling paradigm; AV, audiovisual spelling paradigm.

We computed the latencies of P3a at Fz and P3b at Pz in the V and AV spelling paradigms. The average latencies of P3b and P3a were shorter in the AV spelling paradigm than in the V spelling paradigm. There was no significant difference in the latencies of P3b at Pz between the two paradigms [(AV, V): *t* = −1.949, *P* = 0.067] (Figure 7). The latency of P3a at Fz was significantly shorter in the AV spelling paradigm than in the V spelling paradigm [(AV, V): *t* = −4.001, *P* = 0.001] (Figure 7).

### Offline Accuracy

**FIGURE 8:**
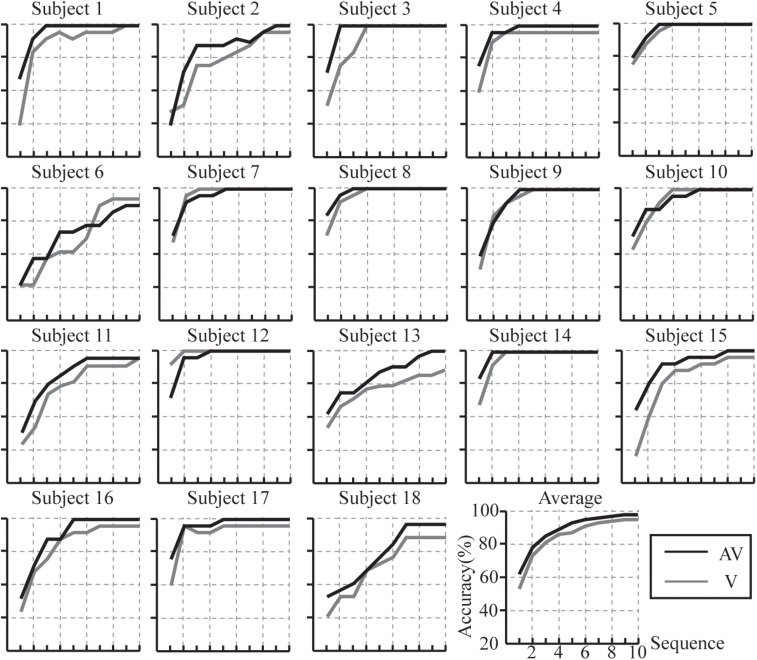
Individual and average accuracies of the P300-speller system with V and AV spelling paradigms for the 18 subjects.

According to the results of the *r*^2^ values and ERP analysis, we selected the feature vector for classification as 40 × 22 (40 represents the sample points between 0 and 800 ms in which there was difference for ERP amplitudes between target and non-target stimuli, and the amplitude of ERPs and latencies of P3a also differed between V and AV spelling paradigms; 22 represents channels F7, F3, Fz, F4, F8, FT7, FC3, FCz, FC4, FT8, C3, Cz, C4, CP3, CPz, CP4, P3, Pz, P4, PO3, POz, and PO4). The individual and average accuracies of the AV and V spelling paradigms for the 18 subjects with different superposition times are shown in Figure 8. The average accuracies were higher in the AV spelling paradigm than in the V spelling paradigm at each superposition. The best results, the accuracy of 100% at two superpositions, were found for subject 3 and subject 14 in the AV spelling paradigm. In this paradigm, the average superposition time was 3.83 for 12 subjects when accuracies reached 100%. In the V spelling paradigm, the average superposition time was 3.63 for eight subjects when accuracies reached 100%.

Accuracies at each superposition between the V and AV paradigms were compared (Table 1). There were significant differences between the V and AV spelling paradigms when superposing from 1 to 10 times (*P* < 0.05), except for superposing 3, 4, 7, and 8 times. However, the accuracies of the AV spelling paradigm had an increasing trend compared with those of the V spelling paradigm at three, four, seven, and eight superpositions (*P* = 0.06). FDR correction was performed for the results.

**TABLE 1:** *T*-test results of accuracies at each superposition time between the V and AV paradigms.

**(V, AV)**	**Superposition times**									
	
	**1**	**2**	**3**	**4**	**5**	**6**	**7**	**8**	**9**	**10**
*t*	−3.205	−2.593	−2.25	−2.001	−3.111	−3.002	−2.097	−2.06	−2.557	−2.557
*p*	**0**.**03**	**0**.**04**	0.06	0.06	**0**.**03**	**0**.**03**	0.06	0.06	**0**.**04**	**0**.**04**

*The bolded values (*P* < 0.05) represent there were significant difference for accuracy between V and AV spelling paradigms.*

**FIGURE 9:**
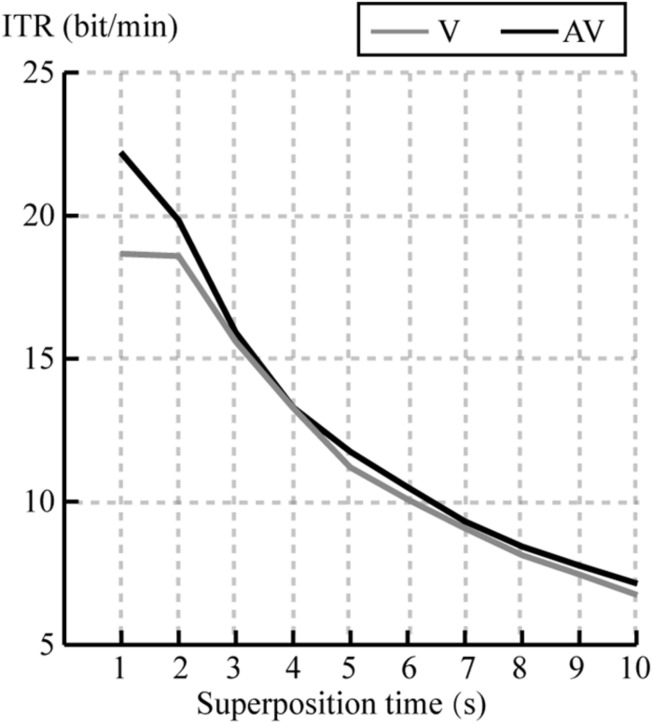
Mean ITR of all subjects at each superposition time for V and AV spelling paradigms.

We compared the ITR at each superposition time for all subjects between V and AV spelling paradigms. Figure 9 shows the average ITR at each superposition time. Average ITR of AV was larger than that of V at all superposition times. A paired *t*-test showed significant differences in ITR between V and AV at superposing 1, 2, 5, 6, and 10 times (*P* < 0.05), as shown in Table 2. The result was corrected by FDR.

**TABLE 2:** Comparison of ITRs at each superposition time between the V and AV paradigms by *T*-tests.

	**Superposition times**									
	
**(V, AV)**	**1**	**2**	**3**	**4**	**5**	**6**	**7**	**8**	**9**	**10**
*t*	−2.863	−2.493	−1.962	−1.773	−3.008	−2.897	−1.597	−1.576	−2.089	−2.523
*p*	**0.04**	**0.049**	0.06	0.08	**0.04**	**0.04**	0.1	0.1	0.08	**0.049**

*The bolded values (*P* < 0.05) represent there were significant difference for ITR between V and AV spelling paradigms.*

## Discussion

### ERP Analyses

In this study, we proposed a novel audiovisual P300-speller system based on temporal, spatial, and semantic congruence. We assessed grand-averaged ERP waveforms elicited by target stimuli in the AV and V spelling paradigms and analyzed the differences in the waveforms of the ERPs elicited by target Trials. In addition, we compared the performance of the P300-speller system between the AV and V spelling paradigms.

In both spelling paradigms, there was a positive waveform with two peaks elicited by target stimuli between 200 and 500 ms at the frontal, central, and parietal areas (Figure 4). The component with a peak between 200 and 300 ms may be P3a potential. The P3a component occurs after a novel event with more frontal distribution, and its latency is usually between 220 and 400 ms (Polich, 2007). The component with a peak between 300 and 500 ms may be the P3b potential because P3b with a more parietal distribution and longer latency is usually between 280 and 600 ms (Polich, 2007). In addition, a negative waveform was observed at approximately 200 ms in the parietal and occipital areas, and it might be the N200 potential (Figure 4). The N200, around 200 ms in the temporal–occipital area, is related to conscious attention to the stimuli (Folstein and Van Petten, 2008).

We analyzed the difference in ERP amplitudes elicited by target stimuli between the AV and V spelling paradigms. First, the amplitudes of target stimuli were significantly larger in the AV spelling paradigm than in the V spelling paradigm between 60 and 140 ms in the frontal area (Figure 5A). Talsma and Woldorff (2005) observed two enhanced positive waveforms for AV (audiovisual stimuli) compared with A + V (the sum of ERPs elicited by unimodal visual and auditory stimuli), one at approximately 100 ms at the frontal area and the other at approximately 160 ms at the central–medial area when the spatial location of the visual and auditory stimuli was matched, indicating the enhanced audiovisual integration. Similarly, Senkowski et al. (2007) observed a positive ERP from audiovisual integration at approximately 60 ms at frontal area, and Teder-Salejarvi et al. (2002) found an audiovisual integration effect with a positive waveform between 130 and 170 ms in the frontal–central area, suggesting that the top-down spatial attention of the audiovisual stimuli may have enhanced the subjects’ responses to the target (Talsma and Woldorff, 2005). Thus, the increased amplitude in our study at the earlier stage (60–140 ms) in the AV spelling paradigm compared with the V paradigm may be because the feature information of audiovisual stimuli increased the attention of the subjects to the target, which enhanced the audiovisual integration effect.

The second ERP waveform with a significant difference between AV and V spelling paradigms was P3b (Figure 5B). P3b, a sub-component of P300, reflects the cognitive demands during task processing (Polich, 2007), and the P3b amplitude will increase when the cognitive demands increase (Horat et al., 2016; Li et al., 2019). In our audiovisual spelling paradigm, the stimulus included information about the following aspects: first, the stimulus was from two sensory channels; second, the congruence of the spatial location and semantic information resulted in more cognitive demands than did the unimodal visual stimulus when subjects recognized the target stimulus. Therefore, the increased P3b amplitude in the AV spelling paradigm compared with the V spelling paradigm in our study may be due to the increased cognitive demands for the audiovisual stimulus. Our findings are consistent with those in the study by Talsma and Woldorff (2005), in which the audiovisual stimulus elicited a larger P3 amplitude (a positive waveform between 350 and 450 ms) than did the visual stimulus when the spatial position of the visual stimulus and the direction of sound source were closely matched.

The third ERP component with a significant difference was between 700 and 780 ms in the frontal and central areas, and it might be a late positive component (LPC) related to the semantically congruent stimuli. Xie et al. (2018) also observed an enhanced LPC amplitude induced by audiovisual stimuli compared with a unimodal stimulus in the semantically congruent condition at electrodes Fz, Cz, and Pz between 700 and 900 ms. The LPC component has been often observed in studies about semantics and in tasks such as memorizing congruous or incongruous auditory sentences (McCallum et al., 1984), memorizing word lists (Martin and Chao, 2001), and making decisions on congruency (Kounios and Holcomb, 1992). Our findings (increased amplitudes between 700 and 780 ms) are consistent with those in previous studies about semantic processing.

Moreover, we also found waveforms with significant differences in the right temporal at 340–480 and 720–780 ms and in the left temporal at 500–780 ms. The enhanced activation areas may be related to the bimodal audiovisual stimulation. Similarly, Raij et al. (2000) investigated the human brain’s audiovisual integration mechanisms for letters and found that auditory and visual brain activations were integrated in the right temporal around 300 ms and in the left and right superior temporal sulci at a later stage (after 500 ms) in a phoneme and grapheme matching task, and Ghazanfar et al. (2005) also observed an increased neural response at the primary auditory cortex caused by semantically matching stimulation. Notably, we found that the difference in ERP amplitudes in the left temporal area was greater than that in the right temporal area at 680–780 ms. Consistently, Campbell (2008) demonstrated that the processing of audiovisual natural speech was activated in the superior temporal regions, in which activation of the left temporal area is usually higher than that of the right temporal area because speech-reading tends to generate left-lateralized activation. Therefore, our findings are in line with those in previous studies.

Although the amplitudes of P3a in the AV spelling paradigm were smaller than those in the V spelling paradigm in the frontal and central areas, there was no significant difference between these two areas. The latency of P3a at Fz in the AV spelling paradigm was significantly shorter than that in the V spelling paradigm. P3a is associated with attention processing, and the latency is related to the speed of allocating attentional resources, in which shorter latencies are related to superior cognitive performance (Polich, 2007). A study about attention effects on the integration of auditory and visual syllables found that the latency of P3a during an audiovisual attention task was shorter than that during a visual attention task, indicating that letter–speech sound integration helps subjects to respond quickly to stimuli (Mittag et al., 2013). Therefore, the shorter P3a latency in the AV spelling paradigm than in the V spelling paradigm may have resulted from faster responses to audiovisual stimuli than to unimodal visual stimuli.

### Classification Accuracy and ITR of AV and V Spelling Paradigms

We compared and analyzed the classification accuracies and ITRs between the AV and V spelling paradigms. As expected, the average offline accuracies of the AV spelling paradigm were higher than those of the V spelling paradigm at each superposition. Significantly better accuracies were observed at 1, 2, 5, 6, 9, and 10 superposition times in the AV spelling paradigm than in the V spelling paradigm (Table 1). As shown in Table 1, there was an improvement trend at three, four, seven, and eight superpositions, although this improvement was not significant (*P* > 0.05). Studies have shown that more ERPs and ERPs with larger amplitudes contribute to improved classification accuracy of the P300-speller (Kaufmann et al., 2011; Li et al., 2015). In our study, the spatially and semantically congruent audiovisual stimuli elicited larger ERP amplitudes at 60–140, 360–460 (P3b), and 700–800 ms than the unimodal visual stimuli, indicating significant improvement in the accuracies of the P300-speller paradigm. In addition, the latency of the P300 can be used as a measure of information processing speed for cognitive functions and contributes to detection of target stimuli (Yagi et al., 1999). Some psychophysical studies have shown that behavioral responses to audiovisual stimuli are typically faster and more accurate than those to unimodal stimuli alone (McDonald et al., 2000; Teder-Salejarvi et al., 2002). In our ERP analyses, the latency of p3a for the audiovisual stimulus was shorter than that for the visual stimulus, indicating that the processing speed of audiovisual information is faster than that of visual information and the faster processing speed may help improve the classification accuracy.

Information transfer rates are an important index to measure the performance of the BCI system, which depends on both classification accuracy and character output speed, and the speed of character output depends on the length of SOA and the times of stimuli repeating. The increased SOA would result in larger P300 amplitude to improve the classification accuracy (Lu et al., 2013), but would lead to a longer time for character selection. Therefore, classification accuracy and the speed of character selection must be weighted for obtaining higher ITR in the design of the P300-speller paradigm. In our study, the set of SOA (250 ms) was to ensure the stable classification accuracy and the pronunciation integrity for each character, which may bring losses more or less to ITR. We compared ITRs at each superposition time between V and AV spelling paradigm (Figure 9 and Table 2), and the results showed that ITRs at superposing 1, 2, 5, 6, and 10 times were significantly larger for the AV spelling paradigm than for the V spelling paradigm. Future studies are needed to determine how to reduce SOA to improve ITR while ensuring the stability of accuracy and the integrity of character pronunciation to further optimize the performance of the audiovisual p300-speller.

The proposed novel audiovisual P300-speller paradigm significantly improved the performance of the P300-speller compared with the visual-based paradigm. The spatially and semantically congruent audiovisual P300-speller was based on a traditional region flashing paradigm. This paradigm requires time to transition between the group-area and sub-area, which reduces the speed of character spelling. Therefore, further investigations are needed to adjust the transformation time between group-area and sub-area to further improve the ITR of the audiovisual P300-speller.

### Subject Reports

After collecting EEG data from each subject, he/she was asked about his/her comfort with the AV and V spelling paradigms. Fourteen subjects stated that the audiovisual stimuli from the left or right positions helped them focus on the target stimuli, and the auditory stimuli had a certain hint effect when the superposition times increased. Notably, when the spelling paradigm ran into double flashing problems, it was difficult for them to judge whether the stimulus had flashed once or twice in the unimodal visual condition. However, in the audiovisual condition, the subjects could judge the times of stimulus intensification according to the auditory stimulus of the target. The remaining four participants initially felt that the unimodal visual stimulus would make them more focused on the target. However, as the spelling characters increased, they were more comfortable focusing on the target with audiovisual stimuli because the auditory and visual channels hinted each other. This may be one of the reasons why the accuracies were still significantly improved for the AV spelling paradigm compared with the V spelling paradigm when the superposition times increased. Although the subjects’ reports only reflected subjective feelings regarding the spelling paradigm, they have significant implications for the development and improvement of the audiovisual P300-speller paradigm, especially for the application of BCI for patients with neurodegenerative diseases, in which muscular control of the eyes may be impaired or deteriorate over time.

## Conclusion

In this study, we proposed a novel P300-speller paradigm based on audiovisual spatial and semantic congruency to investigate whether spatial and semantic matching of audiovisual stimuli can improve the classification accuracy of visual-based P300-spellers. We found that the novel audiovisual P300-speller had significantly improved performances compared with the visual-based P300-speller. Our findings enhance the versatility of the P300-speller system because it is not only suitable for patients with limited hearing but also for those with impaired or deteriorating vision over time. In addition, subjects’ reports on the comfort of the paradigm are of great value in helping further develop the audiovisual-based P300-speller system.

## Data Availability Statement

All datasets generated for this study are included in the manuscript/supplementary files.

## Ethics Statement

The studies involving human participants were reviewed and approved by the Changchun University of Science and Technology. The patients/participants provided their written informed consent to participate in this study.

## Author Contributions

The manuscript was written with contributions from all authors. All authors have approved the final version of the manuscript. QL and ZL designed the experiments. ZL and JY performed the experiments. ZL and JY analyzed the experimental results. NG and OB checked and verified the experimental results. QL and ZL wrote the manuscript.

## Conflict of Interest

The authors declare that the research was conducted in the absence of any commercial or financial relationships that could be construed as a potential conflict of interest.
